# Hepatitis C Treatment & Management

**Published:** 2014-03-25

**Authors:** D Andronescu, S Diaconu, N Tiuca, RM Purcarea, CI Andronescu

**Affiliations:** * “Carol Davila” University of Medicine and Pharmacy, Gastroenterology Department, Emergency University Hospital, Bucharest; ** “Carol Davila” University of Medicine and Pharmacy, “Dr. Carol Davila” Clinical Nephrology Hospital, Bucharest, Romania

**Keywords:** hepatitis C, interferons, ribavirin, protease inhibitors

## Abstract

Abstract

Combination therapy with pegylated interferon alfa (PEG-IFN alfa) and the nucleoside analogue ribavirin is the current standard of care in patients infected with hepatitis C virus (HCV). Patients with HCV genotype 1 have a much less favorable response to therapy and are treated for 12 months, compared with patients infected with genotypes 2 and 3, in whom a 6-month course of therapy is sufficient.

If viremia is present after 6 months, additional therapy has a negligible benefit, and treatment should be stopped in all patients regardless of the viral genotype. With HIV coinfection, all patients with a response to therapy at the end of 6 months should receive an additional 6 months of combination therapy regardless of the genotype. Patients with acute HCV infection should be treated for 6 months.

The addition of protease inhibitors to the combination of PEG-IFN alfa and ribavirin is becoming the new standard of care for the treatment of chronic HCV infection. Regimens that include a protease inhibitor significantly improve sustained virologic response rates in patients with genotype 1 HCV infection.

**Interferons and Ribavirin**

A major advance in the treatment of chronic hepatitis C was the addition of the oral nucleoside analogue ribavirin to the IFN regimen. As reported in the studies by McHutchison et al and Poynard et al, [**1**,**2**] IFN alfa-2b and ribavirin combination therapy for 6-12 months resulted in sustained eradication rates of 30-40%. However, patients with HCV genotype 1 who were treated for 12 months had a much less favorable response than patients infected with genotypes 2 and 3 who received a 6-month course of therapy.

**PEG-IFN therapy with ribavirin**

The addition of ribavirin to PEG-IFN heralded a new era in the treatment of chronic HCV. The benefits of combination therapy were documented in 3 trials: Manns et al from 2001, [**3**] Fried et al from 2002, [**4**] and Hadziyannis et al from 2004 [**5**].

Manns et al reported a higher SVR rate in patients given higher-dose PEG-IFN alfa-2b plus ribavirin than in patients given lower-dose PEG-IFN alfa-2b plus ribavirin or given IFN alfa-2b plus ribavirin [**3**] (**Fig. 1**). Secondary analyses identified body weight and HCV RNA viral load less than 1 million copies per milliliter as important predictors of SVR (**Fig. 1**).

**Fig. 1:**
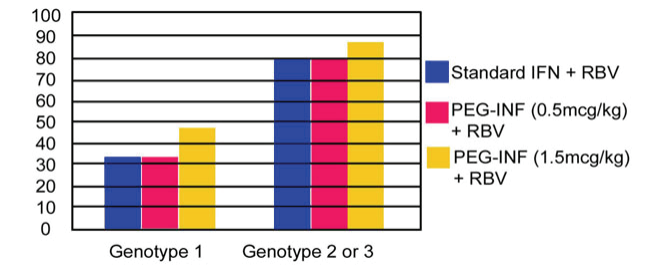
PEG – IFN Alfa 2b + RBV Virologic Response by Genotype (Manns [**3**])

Fried at al found that patients who received PEG-IFN alfa-2a plus ribavirin had a significantly higher SVR rate than patients who received IFN alfa-2b plus ribavirin (56% vs 44%) or PEG-IFN alfa-2a alone (56% vs 29%) [**4**]. The SVR rates for patients with HCV genotype 1 were 46%, 36%, and 21%, respectively, for the 3 regimens.

 Hadziyannis et al reported that in patients infected with HCV genotype 1, 48 weeks of treatment was statistically superior to 24 weeks, and standard-dose ribavirin was statistically superior to low-dose ribavirin [**5**]. In this study, 1311 persons were randomized to PEG-IFN alfa-2a at 180 mcg/wk for 24 or 48 weeks plus a low dose (800 mg/day) or standard weight-based dose (1000 or 1200 mg/day) of ribavirin [**5**]. In patients with HCV genotypes 2 or 3, there were no statistically significant differences in SVR rates in the 4 treatment groups.

 In a study of ribavirin in combination with either PEG-IFN alfa-2b or PEG-IFN alfa-2a for the treatment of chronic HCV infection, Ascione et al reported a higher SVR rate with PEG-IFN alfa-2a than with PEG-IFN alfa-2b (68% versus 54.4%) [**6**].

 In a similar trial, Rumi et al reported that treatment with ribavirin plus PEG-IFN alfa-2a resulted in a significantly higher SVR rate than ribavirin plus PEG-IFN alfa-2b [**7**,**8**].

In a study of patients coinfected with HCV and HIV with compensated cirrhosis, Mira et al found that SVR to PEG-IFN plus ribavirin significantly reduced the incidence of liver-related decompensations and overall mortality [10].

 In conclusion, treatment with PEG-IFN alfa-2a and ribavirin may be individualized by genotype. Patients with HCV genotype 1 require treatment for 48 weeks and a standard dose of ribavirin; those with HCV genotype 2 or 3 seem to be adequately treated with a low dose of ribavirin for 24 weeks [**9**].

 Response to therapy of HCV genotype can now be predicted by identifying the single neoceotide polymorphisms (SNPs) located in the region of interleukin (IL)-28B gene through genome-wide association studies. Patients with CC genotype of the IL-28B have much more favorable response as compared to CT or TT genotype (70% vs 25-30%). Testing for IL-28B genotype is thus a useful tool in the management of patients [**31**].

 Adverse effects

Adverse effects of IFN include the following:

 • Hematologic complications (i.e., neutropenia, thrombocytopenia)

• Neuropsychiatric complications (i.e., memory and concentration disturbances, visual disturbances, headaches, depression, irritability)

 • Flulike symptoms

• Metabolic complications (i.e., hypothyroidism, hyperthyroidism, low-grade fever)

• Gastrointestinal complications (i.e., nausea, vomiting, weight loss)

• Dermatologic complications (i.e., alopecia)

 • Pulmonary complications (i.e., interstitial fibrosis)

Adverse effects of ribavirin include the following:

 • Hematologic complications (i.e., hemolytic anemia)

• Reproductive complications (i.e., birth defects)

• Metabolic complications (i.e., gout)

Growth factors, such as granulocyte-stimulating factor (GSF) and erythropoietin, are frequently used to counteract the adverse hematologic effects of IFN and ribavirin, respectively.

In November 2012, the US Food and Drug Administration (FDA) approved eltrombopag (Promacta), an oral thrombopoietin agonist, for treatment of thrombocytopenia in patients with chronic hepatitis C to allow the initiation and maintenance of IFN-based therapy. The approval was based on results from the phase 3 Endoscopic Ablation Using Light Energy (ENABLE) 1 and 2 trials, which showed eltrombopag significantly reduced the time to the first IFN dose reduction compared with placebo [**11**].

 Because of this, a significant improvement in virologic response was observed in the eltrombopag group compared with placebo.

 In patients who are at risk of depression or who develop depression during treatment, any antidepressant is better than none. Because available evidence suggests that all antidepressants will have an effect.

Fatigue is common in patients with chronic hepatitis C but is poorly associated with biochemical parameters. Sustained response is accompanied by substantial improvement of fatigue [**12**].

Sofosbuvir and ribavirin

In 2 randomized studies in patients with chronic HCV genotype 2 or 3 infection in whom treatment with PEG-IFN and ribavirin was not an option, treatment with sofosbuvir and ribavirin was shown to be effective [**13**,**14**].

In the first study, patients in whom PEG-IFN treatment was not an option were treated with oral sofosbuvir and ribavirin (n = 207) or placebo (n = 71) for 12 weeks. In the second study, patients who had not responded to IFN therapy were treated with sofosbuvir and ribavirin for 12 weeks (n = 103 patients) or 16 weeks (n = 98) [**13**].

In the first study, the rate of sustained virologic response at 12 weeks was 78% with sofosbuvir and ribavirin and 0% with placebo. In the second study, rates of response were 50% with 12 weeks of treatment and 73% with 16 weeks of treatment. Response rates were lower in patients with genotype 3 infection than in patients with genotype 2 infection in both studies. Efficacy was higher among patients without cirrhosis [**13**]. Recently, for HCV genotype 1, Sofosbuvir – based triple therapy including PEG–IFN produced an SYR 12 of 89%.

Protease Inhibitors

 The current available therapies for chronic HCV infection are effective in fewer than 50% of patients with HCV genotype 1. A new class of direct-acting antiviral agents (DAAs) has revolutionized the treatment of HCV genotype 1 infection. These drugs target specific enzymes involved in viral replication. The addition of these new protease inhibitors to pegylated interferon and ribavirin has become the new standard of care for the treatment of chronic HCV infection.

Boceprevir (Victrelis) and telaprevir (Incivek) are HCV NS3/4A protease inhibitors and were approved by the US Food and Drug Administration in May 2011. Each is indicated for treatment of chronic HCV genotype 1 infection in combination with PEG-IFN alfa and ribavirin in adults with compensated liver disease, including cirrhosis, who are previously untreated or who have failed previous interferon and ribavirin therapy.

Boceprevir was evaluated in 2 phase 3 clinical trials with nearly 1,500 patients. In both trials, two thirds of patients receiving boceprevir in combination with peginterferon alfa and ribavirin experienced a significantly increased sustained virologic response (i.e., undetectable HCV RNA level) compared with those taking peginterferon alfa and ribavirin alone [**15**,**16**].

 In a recent double-blind, placebo-controlled trial of 201 patients with HCV genotype-1 who had relapsed or not responded to previous therapy, the addition of boceprevir after 4 weeks of lead-in therapy with PEG2a/R significantly increased the rate of SVR from 21% in the PEG2a/R group to 64% in the BOC/PEG2a/R group (P < .0001) [**17**] . In this study, a ≥1-log10 decline in HCV RNA at treatment week 4 was the strongest independent predictor of SVR to added boceprevir therapy (39% vs 71%).

The safety and effectiveness of telaprevir was evaluated in 3 phase III clinical trials with about 2,250 patients who were previously untreated or who had received prior therapy. In previously untreated patients, 79% of those receiving telaprevir experienced a sustained virologic response (SVR) compared with PEG-IFN alfa and ribavirin treatment alone. SVR for patients treated with telaprevir across all studies and across all patient groups was 20-45% higher than the current standard of care [**18**].

The Protease Inhibition for Viral Evaluation 1 (PROVE1) study demonstrated that the addition of telaprevir to the current treatment regimen improved virologic response to HCV [**19**]. However, the telaprevir groups had a higher rate of discontinued treatment (21%) compared with the placebo group (11%) because of adverse effects, particularly rash.

In another recent study, telaprevir monotherapy for 2 weeks reduced levels of HCV RNA in patients with chronic HCV genotype 2 infections, but had limited activity in patients with HCV genotype 3 [**20**,**21**].

 The chosen treatment regimen takes into consideration prior exposure and response to pIFN and ribavirin presence of cirrhosis and a selected direct antiviral agent (DAAs). (**Fig. 2**), (**Fig. 3**), (**Table 1**)

**Fig. 2:**
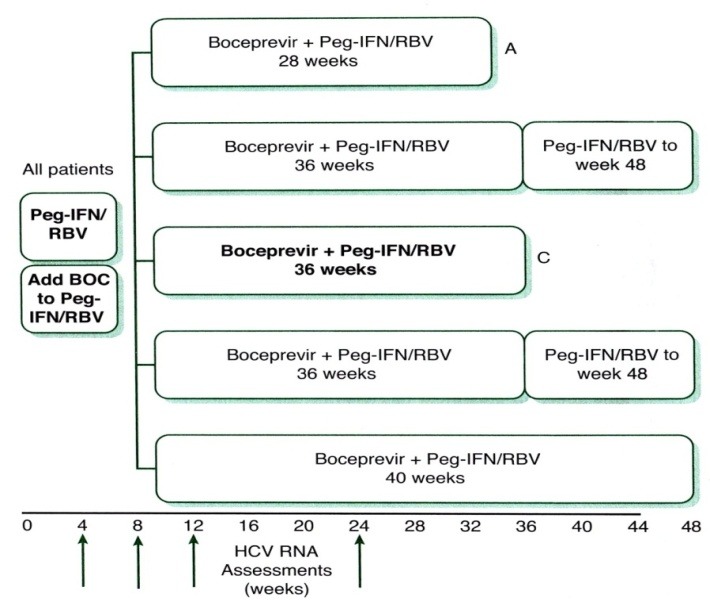
2 Response – guided treatment algorithm for boceprevir. HCV, hepatitis C virus; Peg-IFN/RBV, pegylated interferon/ribavirin. A: Naïve, undetectable HCV RNA at weeks 8 and 24. B: Naïve, detectable HCV RNA at week 8 and undetectable HCV RNA at week 24. C: Experienced, undetectable HCV RNA at weeks 8 and 24. D: Experienced, detectable HCV RNA at week 8 and undetectable HCV RNA at week 24. E: Cirrhosis

**Fig. 3:**
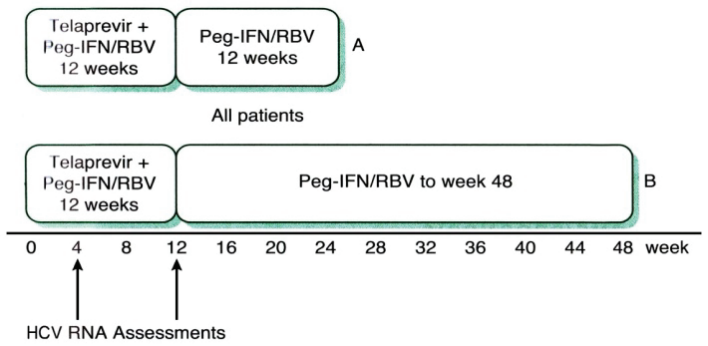
Response – guided treatment algorithm for telaprevir. HCV, hepatitis C virus; Peg-IFN/RBV, pegylated interferon/ribavirin. A: Naïve, undetectable HCV RNA at weeks 4 and 12. B: Naïve, detectable HCV RNA at weeks 4 and/or 12; all experienced patients; naïve, cirrhotic, undetectable HCV RNA at weeks 4 and 12

**Table 1:** Futility Rules for Boceprevir and Telaprevir

Triple therapy with boceprevir should be stopped if the patient has:
HCV RNA results ≥100 IU/ml at week 12
Confirmed, detectable HCV RNA at week 24
Triple therapy with telaprevir should be stopped if the patient has:
HCA RNA results ≥ 1000 IU/ml at weeks 4 or 12
Confirmed, detectable HCV RNA at week 24
HCV, hepatitis C virus.

Newer approaches to hepatitis C therapy involve treatment with 2 direct-acting antiviral agents. In a preliminary study a combination of NS5A replication complex inhibitor daclatasvir (60 mg once daily) and the NS3 protease inhibitor asunaprevir (600 mg twice daily) resulted in a sustained virologic response in patients with HCV genotype 1 infection who had not responded to prior therapy with peginterferon and ribavirin [**24**].

 Nonresponse or Relapse

 For patients who do not achieve an SVR after a full course of PEG-IFN plus ribavirin, retreatment is not recommended, even if a different type of PEG-IFN is used [**4**]. Patients who do not respond to antiviral therapy and who have advanced fibrosis should be screened for hepatocellular carcinoma (HCC) and varices and should be evaluated for liver transplantation if they are appropriate candidates. Patients with mild fibrosis should be monitored without treatment [**4**].

 Hayashi and Kasahara noted that exposure to IFN, irrespective of HCV eradication status, was associated with a reduced incidence of HCC [**22**]. 

 The aim of treating decompensated cirrhotic patients is to achieve sustained viral eradication before liver transplantation in an attempt to prevent recurrent HCV infection. Although viral titers may decrease during treatment and possibly diminish the severity of recurrent HCV infection, complications and successful eradication are less likely in patients with more advanced liver disease [**23**].

Recurrence after liver transplantation

Recurrent HCV infection is universal after liver transplantation, can lead to cirrhosis in 30% of patients within 5 years, and is emerging as the most common cause of retransplantation [**22**]. IFN is contraindicated after organ transplantation because of its high risk of precipitating rejection, in part due to upregulation of the human leukocyte antigen (HLA) system by IFN. Liver transplantation is a possible exception; however, as allograft rejection is uncommon in liver transplant recipients with recurrent HCV infection who are treated with IFN-based therapies.

Samuel et al, found that the combination of IFN alfa-2b plus ribavirin induced an SVR in 21% of transplant recipients with recurrent hepatitis C [**25**]. However, no significant histologic improvement was noted, and antiviral therapy was discontinued in 43% of treated patients because of adverse events (primarily severe anemia).

Despite the low rates of sustained viral eradication in patients with post transplantation HCV recurrence, Narayanan Menon et al identified a subgroup of patients who demonstrated improved fibrosis scores despite failure to eradicate the virus [**25**,**26**]. This suggests that some patients may benefit from maintenance therapy and emphasizes the benefit of performing pretreatment and post treatment biopsies in this group of patients.

Two 2003 pilot studies on the use of PEG-IFN alfa-2b and ribavirin in these patients, by Mukherjee et al [**27**] and Rodriguez-Luna et al,[**28**] found that sustained eradication rates were less than 30%.

Patients with Normal Liver Enzyme Levels

The treatment for these patients remains controversial; because previous studies have demonstrated that, they frequently have mild liver disease, do not tolerate therapy, or can develop new elevations in liver chemistry parameters after starting treatment. Liver biopsy can be valuable in these cases. Hui et al reported that ALT levels and histologic findings are not well correlated and patients can have advanced fibrosis or cirrhosis in the presence of normal liver enzyme levels [**29**].

 Jacobson et al reported that sustained HCV eradication rates in patients with HCV infection and normal ALT values were comparable to those in patients with elevated liver enzyme levels [**30**].

 Patients Using Alcohol or Injection Drugs

 In 1998, Wiley et al reported that significant alcohol use (>40 g alcohol/d in women and >60 g of alcohol/day in men for >5 y) in HCV-infected patients resulted in a twofold to threefold greater risk of liver cirrhosis and decompensated liver disease. In addition, cirrhosis developed more rapidly in alcohol users. Because of the risk that alcohol use poses for rapid liver fibrosis, hepatoma, and deleterious effects on treatment response, complete alcohol abstinence is recommended during treatment.

Practice guidelines from the American Association for the Study of Liver Diseases recommend that HCV treatment not be withheld from patients who use illicit drugs or are on a methadone maintenance program, provided they are willing to maintain close monitoring, including practicing contraception.

Prevention

Currently, no products are available to prevent HCV infection. The development of immunoprophylaxis for this disease is proving difficult.

Patients with hepatitis C should be advised to abstain from alcohol use. Patients with hepatitis C should be advised to use barrier protection during sexual intercourse. Screening high-risk patients and initiating appropriate treatment may decrease the prevalence of cirrhosis and HCC.

 Consultations and Long-Term Monitoring

 Consultation with a gastroenterologist and hepatologist is recommended in the treatment of HCV infection. Consultation with a psychiatrist may be helpful before and during treatment in patients at risk of depression or other psychiatric illnesses. Consultation with a surgeon may be necessary for patients in whom hepatic resection for HCC or liver transplantation is being considered.

 At week 12 of treatment, the patient should be evaluated for an early virologic response by repeating the quantitative HCV RNA and IFN-associated thyroid dysfunction screening. If the HCV RNA level is undetectable or if a greater than 2-log-fold reduction in HCV RNA level is present, therapy should be continued because, according to Fried et al, up to 65% of patients go on developing an SVR [**4**].

Conversely, if an early virologic response is not present, treatment should be stopped, because the chance of developing a sustained response of HCV eradication is less than 3, that the one exception is in the context of clinical trials or treatment of recurrent HCV infection in liver transplant recipients ; improved fibrosis scores have been reported in patients in whom the virus has not been eradicated, thus identifying a subgroup of patients who may benefit from maintenance therapy.

The HCV RNA level should be rechecked 6 months after the completion of treatment; if HCV RNA is detectable, the patient has had a relapse of disease and an alternative treatment should therefore be considered. If HCV RNA is undetectable and test results remain negative, the patient has developed an SVR.

 Patients with HCV infection should be monitored closely for adverse effects as well as response to therapy. Tests to help monitor drug toxicity include the following:

 • Complete blood count with differential

• Renal function testing

 • Liver function tests (including alanine aminotransferase [ALT] level)

 • Thyrotropin level

 While measurement of ALT levels is useful for monitoring the effectiveness of therapy for HCV infection, ALT levels can fluctuate. Consequently, a single value in the reference range does not rule out active infection, progressive liver disease, or cirrhosis. ALT normalization with therapy is not proof of cure.

Patients with cirrhosis should be screened for HCC and esophageal varices. They should also be monitored for the development of decompensated liver disease. Vaccination against hepatitis A virus (HAV) and HBV before or after completing HCV treatment has been recommended.
